# An exploration of social and economic outcome and associated health-related quality of life after critical illness in general intensive care unit survivors: a 12-month follow-up study

**DOI:** 10.1186/cc12745

**Published:** 2013-05-28

**Authors:** John Griffiths, Robert A Hatch, Judith Bishop, Kayleigh Morgan, Crispin Jenkinson, Brian H Cuthbertson, Stephen J Brett

**Affiliations:** 1 Kadoorie Centre for Critical Care Research and Education, John Radcliffe Hospital, Oxford, OX3 9DU, UK; 2 Nuffield Department of Anaesthetics, John Radcliffe Hospital, Oxford, OX3 9DU, UK; 3 Department of Public Health, Old Road Campus, Oxford, OX3 7LF, UK; 4 Sunnybrook Health Sciences Centre, University of Toronto, CA; 5 Centre for Perioperative Medicine and Critical Care Research, Department of Anaesthesia and Intensive Care, Hammersmith Hospital, Imperial College Healthcare NHS Trust, Du Cane Road, London, W12 0HS, UK

## Abstract

**Introduction:**

The socio-economic impact of critical illnesses on patients and their families in Europe has yet to be determined. The aim of this exploratory study was to estimate changes in family circumstances, social and economic stability, care requirements and access to health services for patients during their first 12 months after ICU discharge.

**Methods:**

Multi-center questionnaire-based study of survivors of critical illness at 6 and 12 months after ICU discharge.

**Results:**

Data for 293 consenting patients who spent greater than 48 hours in one of 22 UK ICUs were obtained at 6 and 12 months post-ICU discharge. There was little evidence of a change in accommodation or relationship status between pre-admission and 12 months following discharge from an ICU. A negative impact on family income was reported by 33% of all patients at 6 months and 28% at 12 months. There was nearly a 50% reduction in the number of patients who reported employment as their sole source of income at 12 months (19% to 11%) compared with pre-admission. One quarter of patients reported themselves in need of care assistance at 6 months and 22% at 12 months. The majority of care was provided by family members (80% and 78%, respectively), for half of whom there was a negative impact on employment. Amongst all patients receiving care, 26% reported requiring greater than 50 hours a week. Following discharge, 79% of patients reported attending their primary care physician and 44% had seen a community nurse. Mobility problems nearly doubled between pre-admission and 6 months (32% to 64%). Furthermore, 73% reported moderate or severe pain at 12 months and 44% remained significantly anxious or depressed.

**Conclusions:**

Survivors of critical illness in the UK face a negative impact on employment and commonly have a care requirement after discharge from hospital. This has a corresponding negative impact on family income. The majority of the care required is provided by family members. This effect was apparent by 6 months and had not materially improved by 12 months. This exploratory study has identified the potential for a significant socio-economic burden following critical illness.

## Introduction

Over 100,000 patients are admitted to intensive care units (ICUs) in the United Kingdom (UK) per year. Over the 5 years following an ICU admission there is an excess mortality for these patients compared to an age and sex matched population [1,2]. In addition to excess mortality, there is now a substantial body of evidence to show that significant numbers of survivors of critical illness experience reduced cognitive function and longer-term physical and psychological impairments [3-6].

Up to two thirds of ICU survivors may experience significant problems with physical and psychological health as well as social functioning [7-10]. However, there is limited rigorous research into the social and economic impact that a period of critical illness imposes on both the patient and their immediate family and to date there have been limited attempts to estimate the magnitude of this issue. In 1994, Covinsky et al. demonstrated that 34% of seriously ill hospitalized patients required considerable care-giving assistance from a family member in the 12 months following hospital discharge [11]. In 20% of cases, a family member had to leave work and overall a third of families reported a loss of the major source of income. In 2002, Swoboda and colleagues reported the long-term effects on patients' families after a prolonged stay in a surgical ICU [12]. Almost 60% of responding families provided a moderate or large amount of care-giving between 1 and 9 months after hospital discharge. Just under half had to leave work after 1 month, and more than a third of families had lost savings after 1 year; families moved to a less expensive home, delayed educational plans, or delayed medical care for another family member [12]. A Canadian study, which consisted predominantly of previously healthy and relatively young patients (median age of 43), reported that only 49% had returned to work 1 year after discharge from and ICU, increasing to 73% at 5 years. The reported return to work was mostly driven by economic necessity and difficulties in obtaining state financial support [13]. In the UK, extensive experience of talking with survivors of critical illness and their families [14], suggests that the socio-economic consequences of critical illness similarly extends beyond the individual to impact upon the health and social care systems. Potentially the incomplete physical and non-physical recovery from a period of critical illness experienced by some has an impact on the resumption of independent living and employment; this situation, plus any consequential requirement for care, may jeopardize their wider family social and economic stability.

Thus, the aims of this exploratory study of a representative sample of UK survivors of adult, general intensive care treatment were to: (1) explore the use of a novel questionnaire set; (2) estimate changes in family circumstances and social and economic stability; (3) assess what additional assistance (general care with living and specific health care) patients believed they required and whether these requirements had been delivered and (4) place the findings in the context of the health-related quality of life (HR-QoL) during the year of follow-up.

## Materials and methods

This study was conducted by members of the Intensive Care After Care Network [15], a grouping of health-care professionals with an interest in understanding and improving the long-term outlook for survivors of critical illness, centered around hospitals that provide specific ICU follow-up clinics.

### Participants

This prospective cohort study recruited patients admitted to the ICUs of 22 UK hospitals (11 teaching hospitals and 11 district general hospitals) from 31 August 2008 until 28 February 2010. This multi-center study was approved by an appropriate multi-center research ethics committee (Ref 08/H0502/90). All patients that had received at least 48 hours of level 3 dependency care (critical care for multi-system organ failure) were eligible for inclusion [16]. Patients less than 16 years old, prisoners, those whose primary abode was outside the UK or those who did not consent were excluded. Written informed consent was obtained from patients in the interval between ICU discharge and discharge from the hospital (a minority of patients were also recruited at a routine outpatient visit to a post-ICU follow-up clinic). If a patient lacked capacity then a personal legal representative gave assent for a future written approach from the study office once capacity had been regained. Written consent was sought at 6 months following discharge; patients not responding were excluded from the study.

### Study design

This was a self-completed questionnaire-based study (the study questionnaire booklet forms are presented in Additional file 1). In outline, the booklet comprised the EQ-5D [17], EuroQol Visual Analogue Scale (EQ-VAS) [17], Short Form 36 Version 2 (SF-36v2; licensed *pro bono *from QualityMetric Inc, Lincoln, RI, USA) [18] and a novel question set designed to determine changes in family circumstances, socio-economic stability and care requirements. There was no prohibition on family members or carers helping with survey completion. The questionnaire booklet was mailed to consenting patients at 6 and 12 months after ICU admission together with an accompanying letter. In the case of a non-response, a second questionnaire booklet was mailed. In the event of non-return the protocol allowed the study team to make one telephone call. Patients who expressed a wish not to take part, or those not responding after a telephone call, were excluded from further contact.

For those patients who had initially returned the questionnaire booklet at 6 months, and remained alive, but had failed to respond at 12 months, a protocol amendment was sought and approved by the research ethics committee to allow the patient's primary care physician to be contacted. Death was determined using the NHS Strategic Tracing Service (NSTS). Primary care physicians were contacted after two failed mailing attempts and no response to a home phone call (14-15 months post-ICU discharge). Each primary care physician was asked to clarify whether the patient had died, changed address or had been admitted into a long-term care facility.

### Study materials

Patients were first asked to complete the EQ-5D (minus the visual analogue scale) relating to their health state prior to ICU admission. The questionnaire booklet then included a further EQ-5D, EQ-VAS, SF-36v2 and our novel socio-economic question set to be completed with respect to status at the time of receipt [19-21]. The EQ-VAS is a self-rated health status using a visual analogue scale graduated from 0 (the worst imaginable health state) to 100 (the best imaginable state) [17].

The SF-36 is a comprehensive, generic 36-item questionnaire that is used extensively in clinical practice to describe HR-QoL [18]. It has been demonstrated to be an acceptable and reliable tool for use in the ICU population and its use for quality of life assessment following critical illness has been recommended [22,23]. The Physical Component Score (PCS) and Mental Component Score (MCS) were then calculated in the documented manner and standardized against the UK population using a previously validated computerized macro [20,24].

The demographic, social and economic impact question set was developed in conjunction with the Department of Public Health, University of Oxford. The question set was specifically designed to determine changes in family circumstances, social and economic stability, and working lives and to assess what additional assistance (general care with living and specific health care) patients received or believed they required and whether this had in fact been delivered. The demographic questions were adapted from a draft of the Office of National Statistics' questionnaire intended for use during the 2011 national census [25]. A potential list of other questions was derived from previous question sets used in the study of chronic conditions that are associated with an established social, economic and caregiver burden [26,27]. This potential list was reduced to a manageable question set in terms of time to complete, complexity and intrusiveness. The acceptability and face validity of this question set was subsequently assessed by a group of ICU survivors and relatives, who formed part of the UK Intensive Care Society Patient and Relatives Group, as well as a selection of experienced ICU clinical staff.

Information about the acute illness and ICU stay were derived from the Intensive Care National Audit and Research Centre's (ICNARC) Case Mix Programme data for those units contributing to the Case Mix Programme; a similar data set was retrieved manually from non-ICNARC-contributing ICUs.

Returned questionnaires were read using an electronic form reader (Teleform v10, Cardiff and Cambridge, UK) and the data uploaded into an SQL database (MySQL v5, Oracle Corporation, Redwood Shores, CA), which automated the follow-up process. Data was subsequently exported to Microsoft Excel and IBM SPSS v20 for analysis.

### Analysis

The data underwent a quality assurance process and outlying or inconsistent data points were verified with the contributing investigators. All data were assessed for normality, with subsequent analyses performed using t-tests, non-parametric equivalents or analysis of variance.

## Results

**Figure 1:**
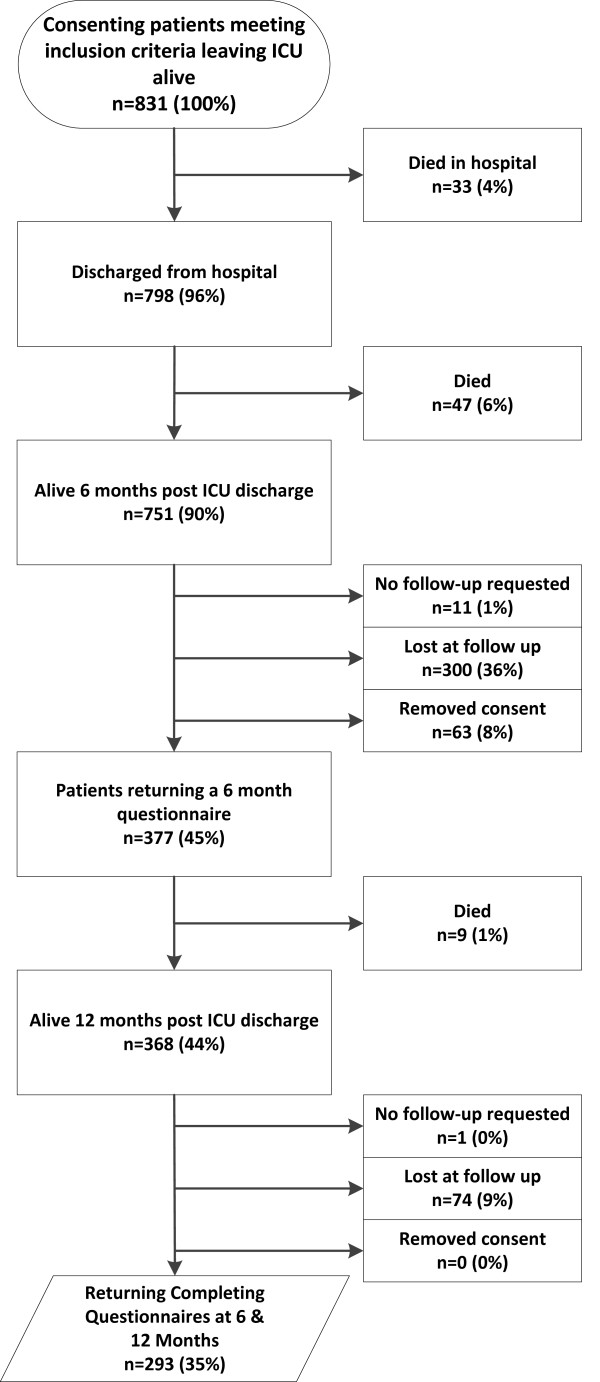
**CONSORT diagram**.

Study enrolment is summarized in the CONSORT diagram (Figure 1). The study coordinating center was notified of 831 potential participants during the enrolment window. Of these, data were collected on 293 patients at both the 6- and 12-month follow-up time points. Demographic details and acute illness characteristics for this subgroup are summarized in Table 1[28].

**Table 1:** Demographics for patients responding at both 6 and 12 months (*n *= 293)

**Sex** ***n*(%)**	**Male** **Female**	**192(66%)** **101(34%)**
Median age (years) (IQR)		62(52-71)
APACHE II *n*(%)	18 ≤ > 18 unrecorded	156(53%) 127(43%) 10(3%)
Median dependency (days) (IQR)	Level 3 care Respiratory support	6(3-12) 4(2-11)
Median length of stay (days) (IQR)	ICU Hospital	8(5-16) 29(17-47)
Type *n*(%)	Medical Surgical emergency Surgical elective Trauma Unknown	173(59%) 59(20%) 30(10%) 30(10%) 1(0%)
ICNARC top 5 diagnoses [28] *n*(%)	Pneumonia, no organism isolated Septic shock Bacterial pneumonia Aortic or iliac dissection or aneurysm Large bowel tumor	37(13%) 29(10%) 21(7%) 20(7%) 5(2%)
Ethnicity *n*(%)	British Irish Other white background No answer African Chinese Pakistani Any other	257(88%) 7(2%) 7(2%) 7(2%) 5(2%) 2(1%) 2(1%) 6(2%)

APACHE II: Acute Physiology and Chronic Health Evaluation II; ICNARC: Intensive Care National Audit and Research Centre; IQR: interquartile range

### Family social and economic impact

Of the responders, 97% reported no change in their relationship status. There was little evidence of a change in accommodation status between ICU discharge and the 6-month and 12-month follow-up time points with the proportion of patients either owning their property outright or with a mortgage (71%) or living in rented accommodation (25%) remaining stable during this period.

**Figure 2:**
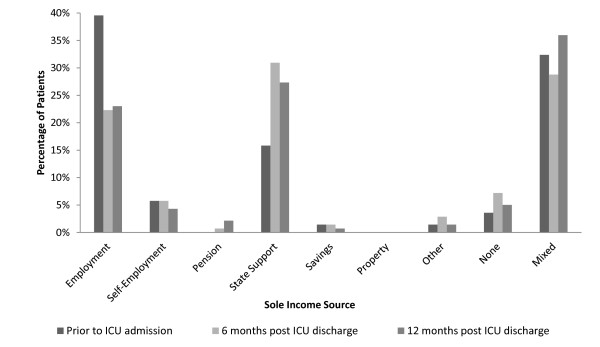
**Sources of income prior to admission and at 6 and 12 months post-ICU discharge**. Mixed income is where the patient has reporting having more than one source of income at any point in time.

Prior to admission 40% of patients reported themselves as being in full or part-time employment. A further 46% were retired, 6% were unemployed, 6% on long-term sick leave and the remainder opted not to disclose. For the purposes of this study, a negative impact in employment status was defined as becoming unemployed, taking early retirement, switching to part-time work or taking long-term sick leave during the study follow-up period. A negative impact on employment was reported by 33% of all patients at 6 months and 28% at 12 months. In parallel, critical illness also impacted on reported family earning source and income. Excluding those patients who were retired prior to ICU admission, there was a demonstrable reduction in reported income from wages or self-employment at 6 months (Figure 2). This reduction predominately occurred in those patients who reported employment as their sole source of income prior to admission; any loss of employment at 6 months largely continued at 12 months and was accompanied by an overall increase in the proportion of patients receiving state financial support. Interestingly, the sum total of individual patients claiming state financial support was less at 12 months in comparison with 6 months. This reduction appears to occur amongst the 32% of patients not retired prior to ICU admission who report receiving their income from at least two separate sources.

**Figure 3:**
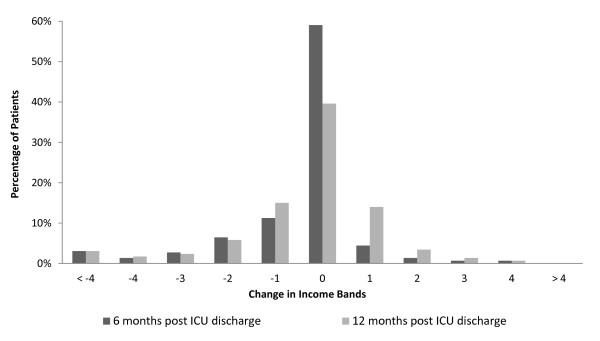
**Changes in financial income brackets**. Income shift at 6 and 12 months compared with pre-admission expressed in terms of changes in income brackets (from question 10 derived from Reference 25).

At 12 months after ICU discharge 45% of all patients reported no change in their monthly family income, whilst 32% reported a reduction and 22% an increase. Figure 3 demonstrates a dominant reduction in reported income bands.

### Requirements for care

**Figure 4:**
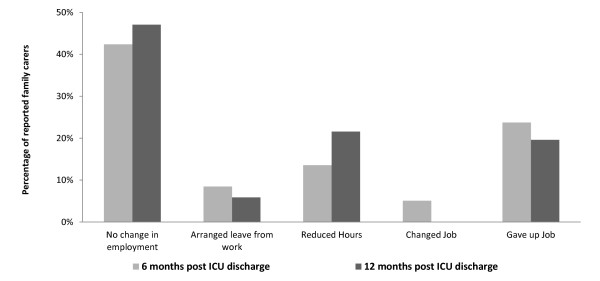
**Changes in employment for family carers**. Changes in employment status amongst families with care requirements.

One quarter (25%) of patients reported themselves in need of help with the activities required for daily living (referred to as care assistance) at 6 months. This had fallen marginally to 22% at 12 months. For those patients receiving care at 12 months following discharge from ICU, 37% needed 0-19 hours a week, with a further 26% needing greater than 50 hours. The majority of this care was provided by family members (80% at 6 months and 78% by 12 months), with approximately 25% of responders receiving care at both 6 months and 22% at 12 months post-ICU discharge. In addition, 51% and 47%, respectively of those families providing care had to make a major adjustment to working life (Figure 4).

In 30 cases at 6 months (10% of the total cohort and 51% of those reporting themselves specifically as needing care), a family member had been given long-term leave, had to leave a job, reduce hours or stop work to provide care. By 12 months, 7 patients no longer declared a family care requirement and four of these family members had been able to return to work after a year. Thus in 8% of all cases, a family member had experienced a significant reduction in employment activity for at least a year.

Of the patients reporting a need for care, 23 patients (31%) at 6 months and 25 (38%) at 12 months had had to spend savings, borrow money, look to charity or remortage/sell their house (or a combination of these) to pay for care.

### Clinical service utilization post-ICU discharge

**Figure 5:**
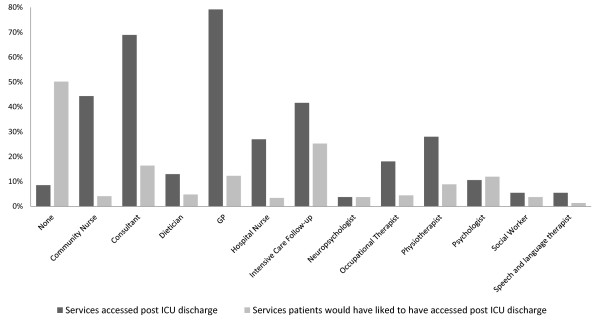
**Services utilized at 12 months post-ICU discharge**. A comparison of the services actually accessed by patients following ICU discharge and services patients feel they would have liked to have accessed following discharge from ICU. GP: general practitioner

Following successful discharge to home, 79% of ICU patients reported attending a clinic appointment with their primary care physician and 44% had seen a community-based nurse (Figure 5); the majority had also been reviewed by a hospital specialist team. The overwhelming majority of these interactions had occurred by the 6-month point. A small number of patients had seen a health professional about social, psychological or emotional issues; this number was exceeded by the number who would have liked to see such an individual.

### Health-related quality of life data

The median scores for overall quality of life using the EQ-5D visual analogue scale was 64 (interquartile range 46 to 80) at 6 months and 66 (interquartile range 44 to 80) at 12 months (not significant *P *= 0.10). The individual domain scores are summarized in Table 2. More patients reported some problems with mobility, compared with their pre-morbid state (58% at 6 months and 54% at 12 months compared with 32% pre-admission); few described themselves as bed-bound. Of the patients, 13% required help with self-care before their acute illness; this had risen to 28% at 6 months and 26% at 12 months. Significantly more patients reported themselves to be experiencing moderate or extreme pain (73% at 6 months and 70% at 12 months compared with 51% pre-admission), or to be moderately or extremely anxious or depressed than before their illness (46% at 6 months and 44% at 12 months compared with 30% pre-admission).

**Table 2:** EQ-5D results for patients responding at 6 and 12 months (*n *= 293)

	Pre-admission %	6 months %	12 months %
**Mobility**			
N: I have no problems in walking about	66%	41%	45%
M: I have some problems in walking about	32%	58%	54%
E: I am confined to bed	2%	1%	1%
**Self-care**			
N: I have no problems with self-care	87%	72%	74%
M: I have some problems washing or dressing myself	12%	26%	25%
E: I am unable to wash or dress myself	1%	2%	1%
**Usual activities**			
N: I have no problems	65%	31%	35%
M: I have some problems	30%	58%	54%
E: I am unable to perform my usual activities	5%	11%	11%
**Pain/discomfort**			
N: I have no pain or discomfort	49%	27%	31%
M: I have moderate pain or discomfort	41%	62%	59%
E: I have extreme pain or discomfort	10%	11%	11%
**Anxiety/depression**			
N: I am not anxious or depressed	70%	54%	56%
M: I am moderately anxious or depressed	26%	41%	37%
E: I am extremely anxious or depressed	4%	5%	7%
**EuroQol UK Tariff**			
Median	0.796	0.691	0.691
25th percentile	0.673	0.516	0.516
75th percentile	1	0.804	0.814
**EQ-5D VAS**			
Median		64	66
25th percentile		46	44
75th percentile		80	80

The SF-36 data demonstrate a marked deficit in all domain scores at both 6 and 12 months when compared with previously published UK norms [24](Table 3). The normalized summary scores, both mental and physical, were similarly reduced compared with UK normal population scores (Table 4).

**Table 3:** SF-36v2 domain scores for patients responding at 6 and 12 months^a^

	6 months	12 months	**Jenkinson et al. **[24]
Physical function	31.66 (15.89)	33.90 (16.66)	87.99 (19.65)
Role physical	31.74 (15.33)	35.23 (15.80)	87.17 (22.01)
Bodily pain	40.08 (12.65)	41.09 (13.11)	78.80 (23.01)
General health	39.76 (12.39)	40.30 (13.14)	71.06 (20.43)
Vitality	43.55 (12.24)	45.33 (12.29)	58.04 (19.60)
Social functioning	40.92 (13.96)	42.95 (13.38)	82.77 (23.24)
Role emotional	42.29 (15.95)	43.10 (15.57)	85.75 (21.18)
Mental health	47.88 (12.48)	48.29 (12.80)	71.92 (18.15)

^a ^Norm-based scores expressed as means (SD)

SF-36v2: Short Form 36 Version 2

**Table 4:** SF-36v2 component scores for patients responding at 6 and 12 months

Physical component score							
				6 months		12 months	
**Parameter**	**Range**	***n***	**%**	**Mean**	**SD**	**Mean**	**SD**

All	196		100%	31.32	14.02	33.74	14.99
Age	≤ 64	120	61%	31.23	14.69	34.58	15.91
	> 64	76	39%	31.46	12.97	32.41	13.41
APACHE	≤ 18	113	60%	31.29	13.79	33.90	15.41
II	> 18	76	40%	30.14	13.66	32.47	14.20
Type	Medical	110	56%	32.48	14.10	34.15	15.14
	Surgical emergency	38	19%	30.19	14.23	33.03	15.27
	Trauma	25	13%	30.42	13.99	35.02	15.11
	Surgical elective	22	11%	27.44	12.51	30.47	13.71

**Mental component score**							

				**6 months**		**12 months**	

**Parameter**	**Range**	***n***	**%**	**Mean**	**SD**	**Mean**	**SD**

All		196	100%	49.17	12.44	49.70	12.78
							
Age	≤ 64	120	61%	47.14	12.87	47.78	13.12
	> 64	76	39%	52.38	11.07	52.73	11.69
APACHE	≤ 18	113	60%	48.10	12.05	48.59	12.96
II	> 18	76	40%	50.35	13.15	50.77	12.81
Type	Medical	110	56%	49.63	12.69	50.03	13.01
	Surgical emergency	38	19%	48.37	12.77	49.38	11.79
	Trauma	25	13%	50.33	11.46	52.04	13.41
	Surgical elective	22	11%	46.81	12.36	45.71	12.78

APACHE II: Acute Physiology and Chronic Health Evaluation II; SF-36v2: Short Form 36 Version 2

### Loss to follow-up

For the 74 patients who were lost to follow-up between the 6- and 12-month follow-up period, additional data was gathered from the respective general practitioners (GPs) for 65 (88%). For 46% of these patients their GP was unaware of any reason that would prevent them from responding to the study mailings. A further 23% were receiving home assistance, 15% had died without the knowledge of the investigators and 4% were in sheltered accommodation or homeless. These patients were not reported in the final cohort of 293 patients.

## Discussion

### Introduction

This is the first UK (or indeed European) study to estimate changes in family circumstance, describe social and economic stability, and quantify the care requirements amongst survivors of ICUs. From this exploratory study we report that approximately one quarter of patients who had received more than 48 hours of level 3 care report being in need of care assistance during the first 12 months after ICU discharge. Family members provide the majority of this care, and half of these family members have made a major adjustment to their own working lives. These ICU patients report reduced HR-QoL compared to population norms and three-quarters experience moderate or extreme pain. Following successful discharge to home, the former ICU patients have a high utilization of a variety of health services. The majority of these patients report attending either their primary care physician or their practice/district nurse.

### Need for assistance with care

One quarter of patients followed up by this study reported themselves in need of assistance with care at 6 months, falling marginally at the 12-month follow-up period. For those patients receiving care at 12 months, a quarter needed greater than 50 hours. Family members provided the majority of this care. Other studies have shown a similar need for care assistance. In 1994, Covinsky et al. demonstrated that a third of seriously ill hospitalized patients required considerable care-giving assistance from a family member in the 12 months following hospital discharge [11]. Swoboda and colleagues [12] reported that almost 60% of responding families provided a moderate or large amount of care-giving. In a further study of informal caregiver burden, caregivers reported spending on average nearly 6 hours a day providing assistance [29]. The highest reported need for care comes from a short-term study by Im et al. of patients who had received at least 48 hours ventilation [30]; 74.8% needed support at 2 months after ICU discharge; the median time spent on providing care was 4 hours/day.

### Family as caregivers

We found that of those families providing care at 6 months and 12 months, around half had to make a major adjustment to their working lives. Van Pelt et al. also showed that a reduction in employment and disruption in lifestyle were common [29]. At 26 months, only 30% of caregivers were employed and 10% indicated that they had stopped working.

It is only over the last few decades that an increased awareness of the potential distress experienced by families of ICU patients has kindled strong interest in family-centered care [31-33]. However, despite these additional stressors we failed to demonstrate either a breakdown of intimate relationships or change in accommodation status during the first 12 months of recovery. The exact reasons for this are unclear. With respect to a major change in accommodation status, one hypothesis would be that perhaps many patients hold a mortgage protection policy and these policies often protect against repossession for an initial 12-month period. Many home loan organizations allow repayment flexibility for a prolonged period after illness; the UK Building Society Association felt that a 1-year follow-up was too short a period to identify repossessions [34].

### Employment and income

This UK study has demonstrated a negative impact on employment and on reported family earning sources and income; this resonates with the North American data in spite of the different social support and economic background. The study by Covinsky et al. reported that a family member had to leave work or make another major life change in 20% of cases [11], and many families reported the loss of the major source of their income and savings. Swoboda report that 44.9% of surviving patients had to quit work after 1 month, and more than 36.7% of families had lost savings after 1 year [12].

We demonstrated an increase in reported use of state financial support at 6 months (Figure 2) and a dominant reduction in reported income bands, for those returning to work. Of the patients reporting a need for care many had to spend savings, borrow money, look to charity or remortage/sell their house to pay for care. In the longer-term follow-up of survivors of Acute Respiratory Distress Syndrome (ARDS), only 49% had returned to work one year after discharge. This had increased to 73% at 5 years. Difficulties in obtaining social financial assistance were reported by many of the survivors [22]. In the study by Im et al., 28.7% of caregivers were working at 2 months but 30.3% had to reduce their working hours (median 16 hours/week) [30]. The economic impact of the critical illness on both patient and family can therefore be significant and long-lasting.

### Health-related quality of life

The HR-QoL data were collected to provide a context for the social and economic data; the SF-36 data demonstrate a marked deficit in all domain scores at both 6 and 12 months and were consistent with previous reports [1,20,35,36]. Conceptually, in this population HR-QoL scores may be related to social and economic burdens. However, evidence in the general literature demonstrates it is not always easy to relate somebody's HR-QoL from their general 'happiness,' financial well-being or social class [37,38].

The EQ-5D data show many need assistance with everyday activities and medical care after they are discharged back to their homes. Family caregivers take on this new and unfamiliar role, and many will experience stress and negative health outcomes themselves. Caregiver distress scores are highest if the people that they are looking after are in obvious pain or discomfort [39]: significantly more of our patients reported themselves to be experiencing moderate or extreme pain than before their illness.

### Access to care and services after ICU discharge

Patients who survive a prolonged stay in an ICU present many challenges to the health-care teams who assume responsibility for their subsequent care. We found that the majority of survivors in our study report visiting their primary care physicians and utilizing community nursing services. It is clear that the foundation of care following critical illness is provided in the community. Thus, primary care physicians are increasingly faced with assuming the responsibility for managing these complex patients.

A third of these patients are referred to specialist medical services and a further third to psychological services. Due to limitations in questionnaire design we cannot exclude a potentially unmet need for specialist care in this patient group. Of particular interest is the access to psychological services - this study demonstrated that 44% of patients report themselves to be moderately or extremely anxious or depressed at 12 months following discharge. However, the number of patients who were able to access psychological services was appreciably lower than the apparent demand. If psychological morbidity is not addressed then return to work may be delayed or prevented [40].

### Strengths and limitations

This study was a comparatively large cohort study [12,29,30]. It is also a multi-center study, which increases its generalizability. We were able to compare services received with services that a patient felt were desirable. Further, we were able to present data on issues relating to patients, families and their caregivers.

The characteristics of the study group are similar to previously published longer-term outcome studies of ICU survivors in the UK and allow comparison with previously published work [1,13,20]. Losses to the study over the first year were due to deaths (10%), withdrawal, and loss to follow-up (36%) and are in keeping with previously published work. Such losses have often been related to the patient's or family member's reports of feeling 'overwhelmed' and reluctant to take on any additional burden of research [31].

This study has several limitations. All follow-up studies are vulnerable to enrolment bias. Patients were consented during the final days of their hospital admission or in an ICU follow-up clinic. This process has clear pragmatic advantages but also includes a potential bias towards those who are more physically or mentally able at this relatively early stage in their recovery.

As with any follow-up study the loss-to-follow-up rate may limit the generalizability of any results. We were unable to record the full characteristics of those patients who opted not to consent or withdrew during follow-up, but where data are available these are presented in Table 5. It is conceivable that the personal nature of some of the questions discouraged patients from participating and this may have introduced a bias towards those remaining in the study.

**Table 5:** Kruskal-Wallis one-way analysis of variance between responders and lost-to-follow-up^a^

	Consented but no questionnaire returned (*n *= 300)	Six-month questionnaire only returned (*n *= 74)	Completed both questionnaires (*n *= 293)^b^	Significance
Median age	62	54	62	0.030
Median ICU stay (days)	9	8	8	0.675
Median APACHE II	17	15	17	0.653
Median level 3 (days)	6	6	6	0.537

^a ^The significance level is .05

^b ^Used in presented analysis

APACHE II: Acute Physiology and Chronic Health Evaluation II

Limited data were collected on the patients and their family status before ICU admission. As ICU admission is generally an unplanned event, it was necessary to rely on patients' recall of their prior functional status, which may have been subject to recall bias. However, others have used a similar approach to tackle this challenge [20]. The lack of a non-critical illness control or comparator population also represents a difficulty. A number of interventional studies have been able to randomize and thus have control data [41]. However, for observational studies, creating a retrospective control sample matched in terms of age, sex and acute and chronic disease is very challenging and has significant limitations.

This was a targeted exploratory study using a novel written question set. Although reviewed by patient and family groups, the question set had not been formally piloted. Following analysis we have excluded a variety of questions that were found to be inconsistent in terms of their response or application. Whilst we have succeeded in advancing our understanding of the socio-economic and family effects of surviving critical illness, we now recognize the limitations of some questions.

Such limitations include our definitions of care-giving and their relevance to the level of care being provided. A more specific assessment of activities of daily living (ADLs) using standardized instruments, such as the Caregiver Assistance Scale, Care-giving impact scale or Personal Gain Scale [29,42], may be useful although they impose a significant question burden, which may have further negatively impacted response rates. Following analysis it became clear that phraseology was in places misleading. Reference to ICU-specific interventions often caused conflicting responses compared with using critical illness as a reference. Inconsistencies were also apparent with questions relating to marital status, household income and use of state financial support since many patients preferred to state their specific benefit or state relief by name and avoid general descriptors (see Additional files 1 and 2) [43].

As the study was conducted in the UK, data concerning access and provision of state financial/social support and health care may not be relevant elsewhere. Our findings concerning changes in employment status for this general ICU population do echo those of the Canadian ARDS cohort, although our population was around 10 years older and thus retirement was more common [13]. Importantly, outcome cohort studies such as this one cannot prove a causal relationship between critical illness or intensive care stay *per se *and the impact described; effects in individuals may be due to their acute illness or progression of pre-existing disease. Similar effects may possibly be observed in a population of survivors of serious illness who did not spend time in intensive care; future studies should perhaps enroll such populations as comparator groups.

## Conclusions

The results of this exploratory study suggest that many patients surviving an intensive care admission have significant functional disabilities, may face a change in their employment and commonly have a care requirement after discharge from hospital. A significant proportion of those who do return to work do not return to their pre-existing level of income or workload commitment. The majority of the care required is provided by family members. In addition, the reduction in patients who return to employment at 6 months and the corresponding use of state financial support remains fairly static at 12 months. This suggests that those family members who become informal caregivers make adjustments to their working lives, resulting in a reduction in financial status. More positively, we did not observe patterns of intimate relationship breakdown or loss of original home, but we cannot exclude this being a major issue in other populations or occurring at a later date. We would cautiously conclude that in families who were experiencing the greatest disruption, this effect was apparent by 6 months and had not materially improved by 12 months. This exploratory study has demonstrated a significant socio-economic burden following critical illness. As important are the methodological lessons learnt, which will hopefully guide future research and ultimately intervention studies aimed at improving these important outcomes.

## Abbreviations

APACHE II: Acute Physiology and Chronic Health Evaluation II; EQ-VAS: EuroQol Visual Analogue Scale; GP: general practitioner; HR-QoL: health-related quality of life; ICNARC: Intensive Care National Audit and Research Centre; IQR: interquartile range; SF-36v2: Short Form 36 Version 2

## Authors' contributions

SJB, JG and BHC conceived and designed the study. SJB, JG and CJ designed the questionnaire. RAH designed the database and questionnaire analysis system. KM and JB coordinated the study and supervised questionnaire dispatch, retrieval and follow-up. SJB, RAH and JG conducted the analysis and produced the first draft of the manuscript. All authors critically revised the manuscript. All authors have seen and approved the final draft of the manuscript.

## Supplementary Material

Additional file 1 Questionnaire booklet Click here for file

Additional file 2 Critique of questionnaire instrument Click here for file

## References

[B1] Cuthbertson BH Scott J Strachan M Kilonzo M Vale L Quality of life before and after intensive care Anaesthesia 2005 60 4 332 339 10.1111/j.1365-2044.2004.04109.x 15766335

[B2] Wright JC Plenderleith L Ridley SA Long-term survival following intensive care: subgroup analysis and comparison with the general population Anaesthesia 2003 58 7 637 642 10.1046/j.1365-2044.2003.03205.x 12790812

[B3] Broomhead LR Brett SJ Clinical review: intensive care follow-up - what has it told us? Crit Care 2002 6 5 411 417 10.1186/cc1532 12398780 PMC137315

[B4] Griffiths J Gager M Alder N Fawcett D Waldmann C Quinlan J A self-report-based study of the incidence and associations of sexual dysfunction in survivors of intensive care treatment Intensive Care Med 2006 32 3 445 451 10.1007/s00134-005-0048-7 16482394

[B5] Griffiths J Fortune G Barber V Young JD The prevalence of post traumatic stress disorder in survivors of ICU treatment: a systematic review Intensive Care Med 2007 33 9 1506 1518 10.1007/s00134-007-0730-z 17558490

[B6] Jackson JC Hart RP Gordon SM Hopkins RO Girard TD Ely EW Post-traumatic stress disorder and post-traumatic stress symptoms following critical illness in medical intensive care unit patients: assessing the magnitude of the problem Crit Care 2007 11 R27 10.1186/cc5707 17316451 PMC2151890

[B7] Sukantarat K Greer S Brett S Williamson R Physical and psychological sequelae of critical illness Br J Health Psychol 2007 12 Pt 1 65 74 17288666 10.1348/135910706X94096

[B8] Sukantarat KT Burgess PW Williamson RC Brett SJ Prolonged cognitive dysfunction in survivors of critical illness Anaesthesia 2005 60 9 847 853 10.1111/j.1365-2044.2005.04148.x 16115244

[B9] Audit Commission Critical to success. The place of efficient and effective critical care services within the acute hospital 1999 London

[B10] Department of Health Critical care outreach 2003: progress in developing services 2003

[B11] Covinsky KE Goldman L Cook EF Oye R Desbiens N Reding D Fulkerson W Connors AF Lynn J Phillips RS The impact of serious illness on patients' families. SUPPORT Investigators. Study to understand prognoses and preferences for outcomes and risks of treatment JAMA 1994 272 23 1839 1844 10.1001/jama.1994.03520230049037 7990218

[B12] Swoboda SM Lipsett PA Impact of a prolonged surgical critical illness on patients' families Am J Crit Care 2002 11 5 459 466 12233971

[B13] Herridge MS Cheung AM Tansey CM Matte-Martyn A Diaz-Granados N Al-Saidi F Cooper AB Guest CB Mazer CD Mehta S Stewart TE Barr A Cook D Slutsky AS One-year outcomes in survivors of the acute respiratory distress syndrome N Engl J Med 2003 348 8 683 693 10.1056/NEJMoa022450 12594312

[B14] Griffiths JA Barber VS Cuthbertson BH Young JD A national survey of intensive care follow-up clinics Anaesthesia 2006 61 10 950 955 10.1111/j.1365-2044.2006.04792.x 16978309

[B15] I-Canuk: Intensive Care After Care Network http://www.i-canuk.com

[B16] Intensive Care Society Levels of Critical Care for Adult Patients 2009

[B17] EuroQol Group EuroQol - a new facility for the measurement of health-related quality of life Health Policy 1990 16 3 199 208 10109801 10.1016/0168-8510(90)90421-9

[B18] The Health Institute SF-36 Health Survey: Manual and Interpretation Guide 1993 Boston

[B19] Badia X Diaz-Prieto A Gorriz MT Herdman M Torrado H Farrero E Cavanilles JM Using the EuroQol-5D to measure changes in quality of life 12 months after discharge from an intensive care unit Intensive Care Med 2001 27 12 1901 1907 10.1007/s00134-001-1137-x 11797026

[B20] Cuthbertson BH Roughton S Jenkinson D Maclennan G Vale L Quality of life in the five years after intensive care: a cohort study Crit Care 2010 14 R6 10.1186/cc8848 20089197 PMC2875518

[B21] Dolan P Gudex C Kind P Williams A A social tariff for EuroQOL of life: results from a general UK general population survey Centre for Health Economics Discussion Paper 138

[B22] Hayes JA Black NA Jenkinson C Young JD Rowan KM Daly K Ridley S Outcome measures for adult critical care: a systematic review Health Technol Assess 2000 4 24 1 111 11074394

[B23] Chrispin PS Scotton H Rogers J Lloyd D Ridley SA Short Form 36 in the intensive care unit: assessment of acceptability, reliability and validity of the questionnaire Anaesthesia 1997 52 15 23 10.1111/j.1365-2044.1997.015-az014.x 9014540

[B24] Jenkinson C Stewart-Brown S Petersen S Paice C Assessment of the SF-36 version 2 in the United Kingdom J Epidemiol Community Health 1999 53 46 50 10.1136/jech.53.1.46 10326053 PMC1756775

[B25] ONS: How we took the 2011 Census http://www.ons.gov.uk/ons/guide-method/census/2011/how-our-census-works/how-we-took-the-2011-census/index.html

[B26] Heffernan C Jenkinson C Measuring outcomes for neurological disorders: a review of disease-specific health status instruments for three degenerative neurological conditions Chronic Illn 2005 1 2 131 142 17136919 10.1177/17423953050010021001

[B27] Mockford C Jenkinson C Fitzpatrick R A review: carers, MND and service provision Amyotroph Lateral Scler 2006 7 3 132 141 10.1080/14660820600601028 16963402

[B28] Harrison DA Brady AR Rowan K Case mix, outcome and length of stay for admissions to adult, general critical care units in England, Wales and Northern Ireland: the Intensive Care National Audit & Research Centre Case Mix Programme Database Crit Care 2004 8 2 99 111 10.1186/cc2816 15025784 PMC420043

[B29] Van Pelt DC Milbrandt EB Qin L Weissfeld LA Rotondi AJ Schulz R Chelluri L Angus DC Pinsky MR Informal caregiver burden among survivors of prolonged mechanical ventilation Am J Respir Crit Care Med 2007 175 2 167 173 10.1164/rccm.200604-493OC 17068327 PMC1899280

[B30] Im K Belle SH Schulz R Mendelsohn AB Chelluri L Prevalence and outcomes of caregiving after prolonged (≥48 hours) mechanical ventilation in the ICU Chest 2004 125 2 597 606 10.1378/chest.125.2.597 14769744

[B31] Daly BJ Douglas SL Kelley CG O'Toole E Montenegro H Trial of a disease management program to reduce hospital readmissions of the chronically critically ill Chest 2005 128 2 507 517 10.1378/chest.128.2.507 16100132

[B32] Douglas SL Daly BJ Caregivers of long-term ventilator patients: physical and psychological outcomes Chest 2003 123 4 1073 1081 10.1378/chest.123.4.1073 12684296

[B33] Rossi Ferrario S Zotti AM Zaccaria S Donner CF Caregiver strain associated with tracheostomy in chronic respiratory failure Chest 2001 119 5 1498 1502 10.1378/chest.119.5.1498 11348959

[B34] (Paul Broadhead TBSA, private communication)

[B35] Dowdy DW Eid MP Sedrakyan A Mendez-Tellez PA Pronovost PJ Herridge MS Needham DM Quality of life in adult survivors of critical illness: a systematic review of the literature Intensive Care Med 2005 31 5 611 620 10.1007/s00134-005-2592-6 15803303

[B36] Ridley SA Chrispin PS Scotton H Rogers J Lloyd D Changes in quality of life after intensive care: comparison with normal data Anaesthesia 1997 52 3 195 202 10.1111/j.1365-2044.1997.073-az0068.x 9124657

[B37] Chandola T Jenkinson C Social class differences in morbidity using the new UK National Statistics Socio-Economic Classification. Do class differences in employment relations explain class differences in health? Ann NY Acad Sci 1999 896 313 315 10.1111/j.1749-6632.1999.tb08126.x 10681907

[B38] Chandola T Jenkinson C The new UK National Statistics Socio-Economic Classification (NS-SEC); investigating social class differences in self-reported health status J Public Health Med 2000 22 2 182 190 10.1093/pubmed/22.2.182 10912557

[B39] Choi J Donahoe MP Zullo TG Hoffman LA Caregivers of the chronically critically ill after discharge from the intensive care unit: six months' experience Am J Crit Care 2011 20 12 22 10.4037/ajcc2011243 21196567 PMC3052639

[B40] Adhikari NK McAndrews MP Tansey CM Matte A Pinto R Cheung AM Diaz-Granados N Barr A Herridge MS Self-reported symptoms of depression and memory dysfunction in survivors of ARDS Chest 2009 135 3 678 687 10.1378/chest.08-0974 19265087 PMC5233444

[B41] Cuthbertson BH Rattray J Campbell MK Gager M Roughton S Smith A Hull A Breeman S Norrie J Jenkinson D Hernandez R Johnston M Wilson E Waldmann C McDonald A McPherson G Ramsay CR Vale L Pflanz-Sinclair C Wildsmith JA Rose S Williams B Walsh T The PRaCTICaL study of nurse led, intensive care follow-up programmes for improving long term outcomes from critical illness: a pragmatic randomised controlled trial BMJ 2009 339 b3723 10.1136/bmj.b3723 19837741 PMC2763078

[B42] Tansey CM Louie M Loeb M Gold WL Muller MP de Jager J Cameron JI Tomlinson G Mazzulli T Walmsley SL Rachlis AR Mederski BD Silverman M Shainhouse Z Ephtimios IE Avendano M Downey J Styra R Yamamura D Gerson M Stanbrook MB Marras TK Phillips EJ Zamel N Richardson SE Slutsky AS Herridge MS One-year outcomes and health care utilization in survivors of severe acute respiratory syndrome Arch Intern Med 2007 167 12 1312 1320 10.1001/archinte.167.12.1312 17592106

[B43] HMRC: Average exchange rates for the year to 31 March 2009 http://www.hmrc.gov.uk/exrate/exchangerates-0809.rtf

