# Supplementary material for: An exploration of social and economic outcome and associated health-related quality of life after critical illness in general intensive care unit survivors: a 12-month follow-up study
Source: Crit Care. 2013 May 28;17(3):R100. doi: 10.1186/cc12745 (PMC3706775; doi:10.1186/cc12745)
Supplement: Additional file 1 — Questionnaire booklet [file cc12745-S1.PDF]

Additional File 1 — Questionnaire Booklet

Question 1

Are you:

- British
- Irish
- Any other white background (specify)
- White & Black Caribbean
- White & Black African
- Any other mixed background (specify)
- Indian
- Pakistani
- Bangladeshi
- Any other Asian background (specify)
- Caribbean
- African
- Any other black background (specify)
- Chinese
- Any other (specify)

Question 2

Does your household own or rent the accommodation?

- Owns outright
- Owns with a mortgage or loan
- Pays part rent and part mortgage
- Rents your accommodation
- Lives rent free

Question 3

What is your marital status?

- Married
- Divorced
- Separated, but still legally married
- Widowed
- Never married

Question 4
Has your marital status changed as a direct result of admission and recovery from your intensive care unit experience?

- No, my marital status has not changed
- Yes, my marital status has changed
  - Divorced
  - Re-married
  - Separated but still legally married

Question 5
What was your employment status BEFORE the event that led to your intensive care unit admission? Please tick one only

- Full-time employment
- Part-time employment
- Early retirement
- Retired (at statutory age)
- Unemployed/not paid employment
- Long-term sick leave
- Other (specify)

Question 6
Were you receiving any of the following financial support PRIOR to your admission to the intensive care unit? Please tick all that apply

- None, I did not apply for financial support
- Any state benefits and/or direct payments from social services
- Care vouchers
- Other (specify)

Question 7
Has your employment had to change as a result of the event that led to your intensive care unit admission? Please tick one only

- No, my employment has not changed
- Yes, my employment has changed
  - Had to give up paid employment
  - Gone part-time

- Taken early retirement
- On long-term sick leave
- Currently unemployed/not paid employment
- Other (specify)

Question 8
Tick as many boxes as you need to show all the sources of income you had in the 12 months BEFORE your admission to the intensive care unit.

- Earnings, wages, salary, bonuses
- Income from self-employment
- Occupational pensions, state retirement pension
- State benefits such as incapacity benefit, child benefit or tax credits
- Interest from savings or investments
- Rent from property
- Other income
- No source of income during that time

Question 9
Tick as many boxes as you need to show all your CURRENT sources of income.

- Earnings, wages, salary, bonuses
- Income from self-employment
- Occupational pensions, state retirement pension
- State benefits such as incapacity benefit, child benefit or tax credits
- Interest from savings or investments
- Rent from property
- Other income
- No source of income during that time

Question 10
From all the sources of income you ticked in question 8, what was your average MONTHLY income BEFORE your admission to intensive care unit?

- Nil
- £1 to £319
- £320 to £599

- £600 to £919
- £920 to £1,319
- £1,320 to £1,839
- £1,840 to £2,839
- £2,840 or more

For comparison £1 = 1.7138 USD or 1.2042 EURO [43]

Question 11
From all of your sources of income you ticked in question 9, what is your CURRENT average MONTHLY income?

- Nil
- £1 to £319
- £320 to £599
- £600 to £919
- £920 to £1,319
- £1,320 to £1,839
- £1,840 to £2,839
- £2,840 or more

Question 12
Are you receiving care?

- Yes
- No Go to question 20

Question 13
Which of the following caring activities do you require regularly? Please tick all that apply.

- Personal care (such as dressing, washing, eating)
- Physical care (such as moving, lifting)
- Household duties (such as shopping, cleaning, cooking)
- Health-care (such as administering medications, treatments, medical devices)

Question 14
How much time per week do you typically depend upon other people providing care for you?

- 1-19 hours
- 20-34 hours
- 35-49 hours
- More than 50 hours

Question 15
Is your carer your next of kin or a close family member?

- Yes
- No, care is provided by social services Go to question 17
- No, I pay for my own care Go to question 17

Question 16
Which of the following has your next of kin or family member experienced in order to provide your care? Please tick all that apply

- They have NOT had to change their job because of their caring responsibilities
- They have been given leave specifically to carry out their caring tasks
- They have had to reduce their working hours because of their caring responsibilities
- They have had to change their job because of their caring responsibilities
- They have had to give up paid employment because of their caring responsibilities

Question 17
Which, if any, of the following do you receive as financial support for care? Please tick all that apply

- None, I did not apply for financial support Go to question 19
- Any state benefits and/or direct payments from social services
- Care vouchers
- Other (specify)

Question 18
How easy have you or your carer found getting financial support from health and social services?

- I did not apply for any
- Very easy
- Easy
- Difficult
- Very difficult

Question 19
Which of the following have you done to provide for your care? Please tick all that apply

- I have done nothing to provide for my care
- Used savings
- Taken out loan/ credit cards
- Re-mortgaged house
- Sold house
- Done extra work
- Applied for money from a charity
- Other (specify)

Question 20
Since your discharge from hospital, who, if anyone, have you seen? Please tick all that apply

- I have not seen anyone about my intensive care unit admission
- Community Nurse/Specialist nurse
- Consultant/ Senior Hospital Doctor
- Dietician
- GP
- Hospital nurse/Specialist nurse
- Intensive Care Follow-up clinic/team
- Neuropsychologist (to assess thinking skills)
- Occupational Therapist (OT)
- Physiotherapist
- Psychologist/ counsellor (for emotional support)
- Social Worker
- Speech and language therapist
- Other (specify)

Question 21

Since your discharge from hospital, who, if anyone, have you seen specifically about your intensive care admission? Please tick all that apply.

- I have not seen anyone about my intensive care unit admission
- Community Nurse/Specialist nurse
- Consultant/ Senior Hospital Doctor
- Dietician
- GP
- Hospital nurse/Specialist nurse
- Intensive Care Follow-up clinic/team
- Neuropsychologist (to assess thinking skills)
- Occupational Therapist (OT)
- Physiotherapist
- Psychologist/ counsellor (for emotional support)
- Social Worker
- Speech and language therapist
- Other (specify)

Question 22

Since your discharge from hospital, who, if anyone, would you have LIKED to have seen specifically about your intensive care admission? Please tick all that apply.

- I have not seen anyone about my intensive care unit admission
- Community Nurse/Specialist nurse
- Consultant/ Senior Hospital Doctor
- Dietician
- GP
- Hospital nurse/Specialist nurse
- Intensive Care Follow-up clinic/team
- Neuropsychologist (to assess thinking skills)
- Occupational Therapist (OT)
- Physiotherapist
- Psychologist/ counsellor (for emotional support)
- Social Worker
- Speech and language therapist
- Other (specify)
