# Supplementary material for: An exploration of social and economic outcome and associated health-related quality of life after critical illness in general intensive care unit survivors: a 12-month follow-up study
Source: Crit Care. 2013 May 28;17(3):R100. doi: 10.1186/cc12745 (PMC3706775; doi:10.1186/cc12745)
Supplement: Additional file 2 — Critique of questionnaire instrument [file cc12745-S2.PDF]

Additional File 2 — Critique of questionnaire instrument - Questionnaire 4

Question 1
Ethnic origin- worked well in our population but may need adapting for studies outside the UK

Question 2
Accommodation- may need adaptation where there may be nomadic or homeless people.

Question 3
Marital status- this question did not have the option for people to describe that they were not married but living together in an intimate relationship or same-sex civil partnership. Some people wrote this on the form. This question needs updating to reflect changing legal situation in many countries.

Question 4
Change in marital status- should really have reflected Question 3 for reclassification if status had changed. This would be more of an issue with very large samples.

Question 5
Employment status- this question should have had the options "self-employed" and "casual employment".

Question 6
State financial assistance- this question will need adapting to local circumstances.

Question 7
Change in employment status- although clear data were returned, question should probably asked "has your employment status changed", to avoid ambiguity for people who may have changed job.

Question 8
Sources of income- although this question did not appear to cause any confusion, this question should have emphasized that it was patient's income- not household income.

Question 9
See Question 8

Question 10
Monthly income- this question was adapted from the UK Office of National Statistics census dataset- this will need to be updated and adapted for current and prevailing income levels. In addition, although this did not appear to cause confusion, disposable i.e.net income should have been specified.

Question 11
See Question 11

Question 12
Receipt of care- although “care is ”defined in Question 13, this should have been defined in this question.

Question 13
Receipt of care- this question worked well.

Question 14
Quantity of care- this question worked well

Question 15
Source of care- although this question worked well, “other” and a text box should probably have been provided.

Question 16
Impact on carers’ employment- seemed to work well

Question 17
Sources of financial support- needs adapting for prevailing social infrastructure. In the UK should have included payout from insurance policies.

Question 18
Ease of obtaining support- straightforward

Question 19
Actions to support care- should probably have asked about help from family.

Question 20
Post hospital discharge healthcare provision- may need local adaptation.

Question 21
Post intensive care discharge healthcare provision- respondents were unable to differentiate this from Question 20- it didn’t work.

Question 22
Professionals patients would have liked to see- phrasing should be tightened to emphasise “who would you have liked to see but did not”. A textbox for reasons would have been useful here.
